# Supplementary material for: Antibiotic-Resistant Bacteria Dissemination in the Wildlife, Livestock, and Water of Maiella National Park, Italy
Source: Animals (Basel). 2023 Jan 27;13(3):432. doi: 10.3390/ani13030432 (PMC9913102; doi:10.3390/ani13030432)
Supplement: Supplementary file 1 [file animals-13-00432-s001.zip › animals-2160645-supplementary.pdf]

animals

MDPI

Supplementary Table S1. Details of oligonucleotides and PCR protocols used for the detection of antimicrobial resistance genes.

Primer	Sequence 5′- 3′	Size (bp)	References
Mcr-1 Fw	AGTCCGTTTGTTCTTGTGGC	320	Rebelo et al. 2018
Mcr-1 Rev	AGATCCTTGGTCTCGGCTTG		
Mcr-2 Fw	CAAGTGTGTTGGTCGCAGTT	715	
Mcr-2 Rev	TCTAGCCCGACAAGCATACC		
Mcr-5Fw	ATGCGGTTGTCTGCATTTATC	207	Jousset et al. 2019
Mcr-5 Rev	TCATTGTGGTTGTCCTTTTCTG		
Mcr-4 Fw	ATTGGGATAGTCGCCTTTTT	487	Carattoli et al. 2013
Mcr-4 Rev	TTACAGCCAGAATCATTATCA		
Mcr-3 Fw	AAATAAAAATTGTTCCGCTTATG	542	Yin et al. 2017
Mcr-3 Rev	AATGGAGATCCCCGTTTTT		
blaTEM F	CCGTGTCGCCCTTATTCCC	780	Kikuvi et al. 2010
blaTEM R	GCCTGACTCCCCGTCGTGT		
IMP-F	GGAATAGAGTGGCTTAAYTCTC	232	Hatrongit et al. 2018
IMP-R	GGTTTAAYAAAACAACCACC		
OXA-48F	GCGTGGTTAAGGATGAACAC	438	
OXA-48R	CATCAAGTTCAACCCAACCG		
NDM-F	GGTTTGGCGATCTGGTTTTC	621	
NDM-R	CGGAATGGCTCATCACGATC		
KPC-F	CGTCTAGTTCTGCTGTCTTG	790	
KPC-R	CTTGTCATCCTTGTTAGGCG		
blaCTX-M F	ATGTGCAGYACCAGTAARGTKATGGC	593	Hasman et a. 2005
blaCTX-M R	TGGGTRAARTARGTSACCAGAAYCAGCGG		
blaCMY-2 F	GCACTTAGCCACCTATACGGCAG	758	
blaCMY-2 F	GCTTTTCAAGAATGCGCCAGG		
blaSHV F	TTATCTCCCTGTTAGCCACC	797	Arlet et al. 1997
blaSHV R	GATTTGCTGATTTCGCTCGG		
blaCMY-1 F	ATGCAACAACGACAATCC	1085	Kim et al. 1998
blaCMY-1 R	TTGGCCAGCATGACGATG		
Tet (A) Fw	GTAATTCTGAGCACTGT	954	Kikuvi et al. 2010
Tet (A) Rev	CCTGGACAACATTGCTT		
Tet (B) Fw	ACGTTACTCGATGCCAT	1170	
Tet (B) Rev	AGCACTTGTCTCCTGTT		
Tet C Fw	AACAATGCGCTCATCGT	1138	
Tet C Rev	GGAGGCAGACAAGGTAT		
Tet(L) Fw	ATAAATTGTTTCGGGTCGGTAAT	1077	Trzcinski et al. 2000
Tet(L) Rev	AACCAGCCAACTAATGACAATGAT		
Tet(M) Fw	ACAGAAAGCTTATTATATAAC	171	Aminov et al. 2001
Tet(M)Rev	TGGCGTGTCTATGATGTTCAC		

Tet(K) Fw	TTATGGTGGTTGTAGCTAGAAA	382	Gevers et al. 2003
Tet(K) Rev	AAAGGGTTAGAAACTCTTGAAA		
VanD_F1	TGGAATCACAAAATCCGGCG	311	Nomura et al. 2018
VanD_R2	TWCCCGCATTTTTCACAACS		
VanM_F1	GGCAGAGATTGCCAACAACA	425	
VanM _R1	AGGTAAACGAATCTGCCGCT		
VanC2_F1	GCAAACGTTGGTACCTGATG	523	
VanC2_R4	GGTGATTTTGGCGCTGATCA		
VanB_F1	GATGTGTCGGTAAAATCCGC	640	
VanB_R1	CCACTTCGCCGACAATCAAA		
VanA_F1	GCAAGTCAGGTGAAGATGGA	721	
VanA_R1	GCTAATACGATCAAGCGGTC		
VanC1_5	GTATCAAGGAAACCTCGCGA	836	
VanC1_6	CGTAGGATAACCCGACTTCC		
VanN_F1	CCTCAAATCAGCAGCTAGTG	941	
VanN_R1	GCTCCTGATAAGTGATACCC		
vanG FW	CGGTTGTGCCGTACTTGGC	810	McKessar et al., 2000
vanG REV	GGGTAAAGCCATAGTCTGGGGC		
Cfr_fw	TGAAGTATAAAGCAGGTTGGGAGTC	746	Bender et al. 2019
Cfr_rev	AACCATATAATTGACCACAAGCAGC		
optrA_fw	TACTTGATGAACCTACTAACCA	422	
optrA_rev	CCTTGAACTACTGATTCTCGG		
poxtA fw	AAAGCTACCCATAAAATATC	533	
poxtA rev	TCATCAAGCTGTTCGAGTTC		
cfr(B) fw	TGAGCATATACGAGTAACCTCAAGA	293	Lee et al. 2017
cfr(B) rev	CGCAAGCAGCGTCTATATCA		
cfr(D) fw	AGAAGTCGCAACAAGTGAGGA	595	Ruiz-Ripa et al., 2020
cfr(D) rev	GCAACTGCATGAGTCAAAGAA		
Vat D F	TCCAGCTAACATGTATGGCG	271	Shaw et al., 2018
Vat D R	GCTCAATAGGACCAGGTGTA		
vgaA F	AGTGGTGGTGAAGTAACACG	659	
vgaA R	CTTGTCTCCTCCGCGAATAC		
vgaB F	TGACAATATGAGTGGTGGTG	576	
vgaB R	GCGACCATGAAATTGCTCTC		
vgbB F	CAGCAGTCTAGATCAGAGTGG	728	
vgbB R	CATACGGATCCATCTTTTCC		
msrC F	AAGGAATCCTTCTCTCTCCG	343	
msrC R	GTAAACAAAATCGTTCCCG		
vgbA F	TACAGAGTACCCACTACCGA	569	
vgbA R	TCAATTCCTGCTCCAGCAGT		
ermB F	CATTTAACGACGAAACTGGC	424	
ermB R	GGAACATCTGTGGTATGGCG		

vatE F	ACTATACCTGACGCAAATGC	511	
vatE R	GGTTCAAATCTTGGTCCG		
ermTR FW	GCTATAGAGATTGATGAAGG	152	Davignon et al., 2000
ermTR REV	CTAATATTGTAGGGAATATTACC		
ermA FW	AAG CGGTAAAACCCCTCTGAG	421	Jensen et al., 1999
ermA FW	TCAAAGCCTGTCGGAATTGG		
ermC FW	CAAACCCGTATTCCACGATT	294	
ermC REV	ATCTTTGAAATCGGCTCAGG		
Pbp1FW	AAACAAGGTCGGACTCAACC	195	Nagai et al., 2001
Pbp1 REV	ATATACATTGGTTTATAGTAAGTT		
Pbp2x FW	CCAGGTTCCACTATGAAAGTG	197	
Pbp2x REV	ATCCCAACGTTACTTGAGTGT		
Pbp2b FW	CCTATATGGTCCAAACAGCCT	147	
Pbp2b REV	GGTCAATTCCTGTCGCAGTA		
gyrA FW	AAATCTGCTCGTGTCGTTGG	349	Ardebili et al., 2015
gyrA REV	GCCATACCTACAGCAATACC		
mefA/E FW	AGTATCATTAATCACTAGTGC	348	Sutcliffe et al.,1996
mefA/E REV	TTCTTCTGGTACTAAAAGTGG		
Sul1 FW	TGGTGACGGTGTTCGGCATTC	789	Turchi et al., 2019
Sul1 REV	GCGAGGGTTTCCGAGAAGGTG		
Sul2 FW	CGGCATCGTCAACATAACC	722	
Sul2 REV	GTGTGCGGATGAAAGTCAG		
Sul3 FW	GAGCAAGATTTTTGGAATCG	792	
Sul3 REV	CATCTGCAGCTAACCTAGGGTTTGGA		
aacC1 FW	GCAGTCGCCCTAAAACAAA	441	Ghotaslou et al., 2017
aacC1 REV	CACTTCTTCCCGTATGCCCAACTT		
aac3 FW	ATATCGCGATGCATACGCGG	877	Hu et al., 2013
aac3 REV	GACGGCCTCTAACCGGAAGG		
armA FW	CCGAAATGACAGTTCCTATC	846	
armA REV	GAAAATGAGTGCCTTGGAGG		
rmtB FW	ATGAACATCAACGATGCCCTC	769	
rmtB REV	CCTTCTGATTGGCTTATCCA		
aacA4 FW	GCTCTTGGAAGCGGGGACGG	300	Sacha et al., 2012
aacA4 REV	TCGCTCGAATGCCTGGCGTG		
aphA6 FW	CATTTGCGGGGTTTTTAATG	837	Aris et al., 2019
aphA6 REV	TTAGATAATGCTTGGAATCA		
rmtC FW	CGAAGAAGTAACAGCCAAAG	1000	Wangkheimayum et al., 2020
rmtC REV	GCTAGAGTCAAGCCAGAAAA		
rmtF FW	GCGATACAGAAAACCGAAGG	589	
rmtF REV	ACCAGTCGGCATAGTGCTTT		

Supplementary Table S2. Table summarizes the resistant bacteria isolated from environmental and fecal samples of wild and domestic animals.

AB	Number of resistant isolates													
	Escherichia coli **(n=40)**	*Enterococcus faecalis* **(n=11)**	*Enterococcus faecium* **(n=12)**	*Enterococcus casseliflavus* **(n=4)**	*Enterococcus gallinarum* **(n=15)**	*Enterococcus hirae* **(n=6)**	*Aeromonas sobria* **(n=1)**	*Enterobacter cloacae complex* **(n=2)**	*Ps. mendocina* **(n=2)**	*Morg. morganii* **(n=1)**	*Str. allactolyticus* **(n=1)**	*Str. mutans* **(n=1)**	*Str. thoraltensis* **(n=1)**	*Str. uberis* **(n=1)**
AMP	3(7.5%)	0	0	0	0	0	0	0	0	0	0	1	0	1
TZP/ TAZ	0	-	-	-	-	-	1	1	0	0	-	-	-	-
BEN	-	-	-	-	-	-	-	-	-	-	0	1	0	1
CTZ	1(2.5%)	-	-	-	-	-	0	1	1	0	-	-	-	-
CTX	0	-	-	-	-	-	0	1	1	0	1	1	1	1
CRO	-	-	-	-	-	-	-	-	-	-	1	1	1	1

ETP	0	-	-	-	-	-	0	0	2	0	-	-	-	-
MRP	1(2.5%)	-	-	-	-	-	0	2	0	0	-	-	-	-
AMK	0	-	-	-	-	-	0	0	0	0	-	-	-	-
CLIN	-	-	-	-	-	-	-	-	-	-	1	1	1	1
ERY	-	-	-	-	-	-	-	-	-	-	-	-	1	1
GEN	0	0	0	0	0	0	0	0	0	0	0	0	0	0
KAN	-	0	0	0	0	0	0	0	0	0	-	-	-	-
STM	-	0	0	0	0	-	0	0	0	0	-	-	-	-

CPFX	0	0	0	0	0	0	0	0	0	0	-	-	-	-
TGC	0	0	0	0	0	0	0	0	0	1	0	1	0	1
NIT	0	0	0	0	0	0	0	0	0	1	-	-	-	-
CS	10(25%)	-	-	-	-	-	0	0	0	0	-	-	-	-
TET	6(15%)	0	-1(9%)	0	3(20%)	0	0	0	0	0	0	0	0	0
SXT	2(5%)	-	-	-	-	-	0	0	0	0	0	0	0	0
LVX	-	0	0	0	0	0	-	-	-	-	0	0	0	0
LIN	-	0	1(9%)	0	6(40%)	0	-	-	-	-	0	1	0	1

DAP	-	0	0	0	0	0	-	-	-	-	-	-	-	-
TEC	-	1(9%)	0	0	0	0	-	-	-	-	0	0	0	0
VAN	-	0	1(9%)	0	0	0	-	-	-	-	0	1	1	0
QD	-	0	1(9%)	0	7(46%)	0	-	-	-	-	-	-	-	-

AMP Ampicillin; AK Amikacin; BEN Benzypenicillin; CS colistin; CLIN Clindamycin; CTX Cefotaxime; CRO Ceftriaxone; ERY Erythromycin; ETP Ertapenem; MER Meropenem; NIT nitrofurantoin; QD quinupristin/dalfopristin; SXT Trimethoprim/sulfamethoxazole; TEIC Teicoplanin; TET Tetracycline; TGC Tigecycline; TZP/TAZ Piperacillin/tazobactam; VAN Vancomycin.
